# Pica is associated with lower willingness to change negative habits of diet and exercise, inadequate lifestyle, and less healthful food consumption in dialysis

**DOI:** 10.3389/fnut.2024.1402625

**Published:** 2024-09-11

**Authors:** Claudia N. Orozco-González, Roxana M. Marquez-Herrera, Fabiola Martín-del-Campo, Laura Cortés-Sanabria, Mariana Villasana-Ballesteros, Alfonso M. Cueto-Manzano

**Affiliations:** ^1^Medical Research Unit of Renal Diseases, Specialties Hospital, Western National Medical Center, Mexican Institute of Social Security, Guadalajara, Mexico; ^2^Nursing School, Autonomous University of the State of Mexico, Toluca, Mexico; ^3^University Center of Health Sciences, University of Guadalajara, Guadalajara, Mexico

**Keywords:** pica, CKD, dialysis, willingness to change, diet, lifestyle, exercise

## Abstract

**Background:**

In dialysis patients, on the one hand unwillingness to change negative lifestyle patterns is associated with worse nutritional status and unhealthy lifestyle, whereas on the other, pica may be highly prevalent. However, it is not known whether pica is associated with unwillingness to change negative lifestyle behaviors, as well as with consumption of different types of foods. This study aimed to investigate this issue.

**Methods:**

This is a cross-sectional study in dialysis patients. Lifestyle was assessed using the self-administered Instrument to Measure Lifestyle Questionnaire (IMEVID). Pica diagnosis was established according to the Diagnostic and Statistical Manual of Mental Disorders, Fifth Edition. A food frequency questionnaire was performed and self-reported willingness to change was determined by a trans-theoretical model staging inventory.

**Results:**

Compared with patients without pica, those with pica (particularly hard pica) had lower willingness to change unhealthy behavior in the case of diet (22% vs. 46% in precontemplation/contemplation stages, respectively) and exercise (43% vs. 62% in precontemplation/contemplation stages, respectively). Patients with hard pica had significantly (*p* < 0.05) lower scores in almost all dimensions of the lifestyle questionnaire than those in the no pica group: diet (23.9 vs. 26.8, respectively), physical activity (5.5 vs. 7, respectively), knowledge of disease (5.7 vs. 6.4, respectively), emotion management (6.6 vs. 8, respectively) and adherence to treatment (13.4 vs. 14.7, respectively), but not in the consumption of tobacco and alcohol. Compared to patients with no pica, those with hard pica ate vegetables and fruits less frequently, and dairy products, fried foods and soda more frequently.

**Conclusions:**

Pica was more frequently observed in patients with lower willingness to change negative habits of diet and exercise, in those who had more unhealthy behaviors in diet, exercise and emotion management dimensions and adherence to treatment, as well as in those who ate less frequently healthful foods and more frequently unhealthy foods.

## 1 Introduction

In patients with end-stage kidney disease (ESKD) on dialysis, unwillingness to change unhealthy lifestyle is associated with worse nutritional status, and with lower consumption of healthy foods and higher intake of unhealthy ones (1–5). Thus, to improve clinical outcomes and decrease complications, it is necessary to recognize the healthy lifestyle non-adherence risks and design tailored strategies to increase motivation and enhance changes of negative lifestyle.

On the other hand, pica, defined as the intake of a substance or object without calories or nutritional properties at least once a month (6), has been the subject of study by multiple disciplines; thus, it has been interpreted in multiple ways as an eating disorder, behavioral problem, symptom of mental illness, expression of nutrient deficiency, abandonment, neglect, poverty or hunger. Etiology of pica is not completely known; sometimes is regarded as a cause and sometimes a consequence (7–9). In patients without kidney disease, pica has been reported to have a 2.35-fold increased odds association with anemia and lower zinc concentrations in a recent meta-analysis (10). In iron deficiency, pagophagia appears to be common in United States, affecting 25% of patients, whereas geophagia is more common in the rest of the world (7); ice seems to be the most common pica item (87%) in non-pregnant women and children (8).

However, in patients with CKD less information is available (11–13), and ice pica was the most frequently found (46%), followed by the soil pica (29%) (14).

Scarce data have been published regarding the association between pica and nutritional variables; in patients without kidney disease, pica was associated with worse nutritional status (15–17); more recently, it has been associated with unmet macronutrient and calorie requirements in patients with ESKD (14). Notwithstanding, it is not known whether pica is associated with the patient's willingness to make positive lifestyle changes and the intake of healthy or unhealthy foods.

Therefore, the present study was aimed to investigate the association between the presence of pica and the willingness to change negative lifestyle behaviors in patients on dialysis, as well as the consumption of different types of foods.

## 2 Methods

This is a cross-sectional study of ESKD patients from a tertiary health-care teaching hospital. Patients in the living donor kidney transplant program were included if they were on dialysis at least 6 months, >18 years old, with any cause of kidney disease and granted their verbal informed consent. They were excluded if had pregnancy, breast feeding, previous transplant, any evidence of infection, or mental illness. Schizophrenia, intellectual disability, organic mental problems, and severe anxious or depressive disorders are specifically excluded before patients enter in our transplant program. This study adhered to the Declaration of Helsinki and was approved by the Local Committee of Research and Ethics (No. R-2016-1301-95).

**Figure 1:**
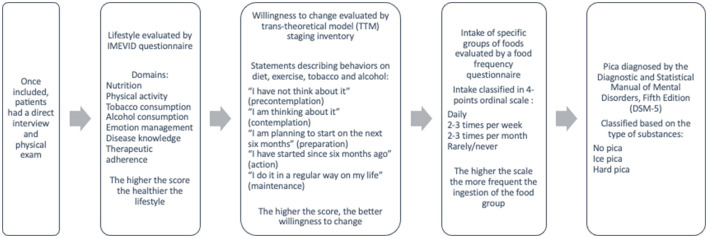
Flow chart of the study procedures.

Socio-demographical and clinical variables were obtained from interview and clinical files; weight was measured just after hemodialysis session. A flow chart showing procedures once the patients were included in the study is shown in Figure 1. Lifestyle was evaluated by the self-administered Instrument to Measure Diabetic Lifestyle Questionnaire (IMEVID) (18), which is made up of 25 items evaluating seven domains: nutrition, physical activity, tobacco consumption, alcohol consumption, emotion management, disease knowledge, and therapeutic adherence. Each item has three possible response options (individualized for each domain), with ratings of 0, 2 and 4 points; total score is 0–100, the higher the score the healthier the lifestyle. IMEVID questionnaire was first developed for diabetes mellitus; thus, the disease knowledge, emotion and adherence domains were modified to consider kidney disease instead of diabetes.

Diagnosis of pica was established according to the Diagnostic and Statistical Manual of Mental Disorders, Fifth Edition (DSM-5) (6) and was classified based on the type of ingested substances as: no pica, ice pica, and hard pica (i.e., starch, soil, clay, grass, paper, or other pica) (6). Of note, only the compulsive ice intake (recognition of intake without thirst) was classified as pica.

A food frequency questionnaire was performed in all patients to evaluate the intake of specific groups of foods; frequency intake answers were classified in a 4-points ordinal scale from never/rarely to daily; the higher the scale the more frequent the ingestion of the food group.

Self-reported willingness to change was determined by selecting a trans-theoretical model (TTM) staging inventory that includes statements reflecting the five stages of change (19). TTM proposes that persons are at varying points of willingness (or readiness) to adopt a specified health-related practice and move about a sequence of stages along a continuum of behavioral change. Participants select the statement that best describes their current behaviors on diet, exercise, tobacco and alcohol from the following 5-points ordinal scale options: (1) “I have not think about it” (precontemplation), (2) “I am thinking about it” (contemplation), (3) “I am planning to start on the next six months” (preparation), (4) “I have started since 6 months ago” (action), and (5) “I do it in a regular way on my life” (maintenance). The higher the score, the better willingness to change (20).

### 2.1 Statistical analysis

Data are shown as mean ± SD or median (25th to 75th percentiles) when dimensional variables had parametric or nonparametric distribution, respectively, or as percentage in the case of nominal variables. Comparisons between groups (no pica, ice pica and hard pica) were performed by ANOVA or Kruskal Wallis tests for dimensional variables, as appropriate: in the case of nominal variables, comparison analysis was done by means of χ^2^ or Fisher exact tests, as appropriate. Cronbach's alpha was calculated to assure internal consistency of the modified lifestyle questionnaire (IMEVID), a value above 0.7 was considered as adequate. A *p* < 0.05 was accepted as significant.

## 3 Results

Four-hundred patients were studied. In the whole sample, mean age was 28 years, 280 (70%) were male, 76% had an unknown cause of kidney disease and only four patients had diabetes mellitus (three in the no pica group and one in the ice pica group). Pica was present in 42% of patients: 46% had ice pica, 29% soil, 14% two substances, 5% red brick, 3% paper, 2% soap, and 1% cattle pasture. Main sociodemographical, clinical and laboratory results are shown in Table 1.

**Table 1:** Comparison of sociodemographic, clinical and biochemical variables according to the type of pica.

**Variable**	**No pica**	**Ice pica**	**Hard pica**
	**(*N* 233)**	**(*N* 78)**	**(*N* 89)**
Age (years)	30 ± 11	27 ± 9	25 ± 7^*^
Male sex (%)	74	63	67
Education years (%)			
<9 years	30	46	57^*^
>9 years	70	54	43
Dialysis vintage (months)	27 ± 16	32 ± 18	36 ± 19^*^
Hemoglobin (g/dL)	10.8 ± 2.2	10.3 ± 2.3	10.4 ± 2.4
Glucose (mg/dL)	85.1± 11.2	84.0± 9.2	86.1 ± 11.0
Creatinine (mg/dL)	11.2 (7–14)	11.4 (9–12)	12.9 (7–15)
Urea (mg/dL)	108 (84–132)	111 (93–143)	116 (78–144)
C-reactive protein (mg/L)	3.0 (3–4.9)	3.4 (3–9.8)^*^	3.0 (3–6.4)
Weight (Kg)	65.5 ± 13.1	64.3 ± 14.0	61.0 ± 12.8^&^
DMS (score)	14.8 ± 3.0	16.0 ± 2.5^*^	16.4 ± 2.6^&^

DMS, Dialysis Malnutrition Score.

^*^p <0.05 vs. no pica.

^&^p <0.05 vs. ice pica.

**Figure 2:**
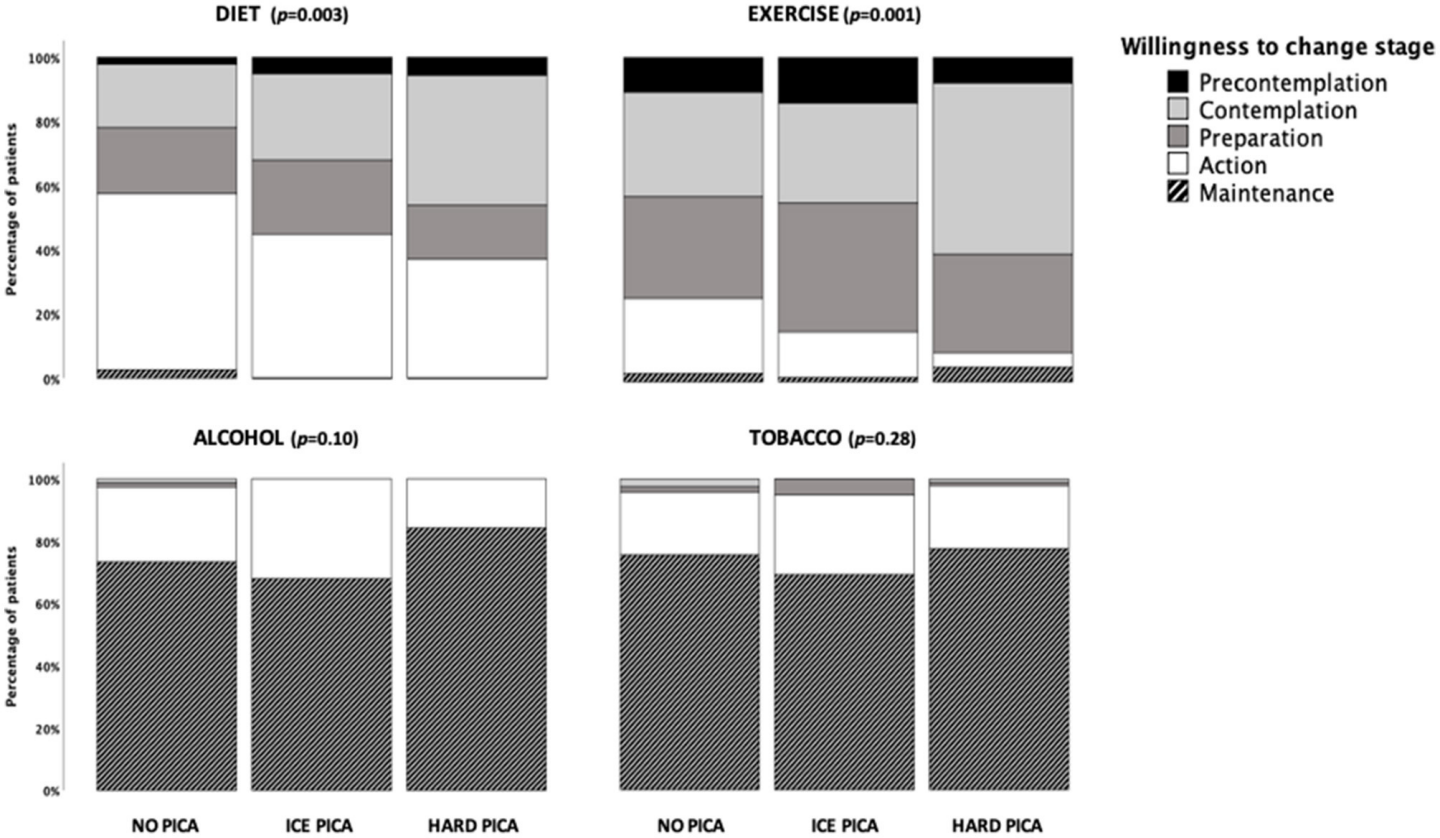
Results of the willingness to change lifestyle behavior according to the presence of pica.

Results of willingness to change lifestyle behavior according to the presence of pica are shown in Figure 2. Patients with pica (particularly hard pica) had lower willingness to change behavior in the case of diet and exercise: a higher frequency were in the contemplation and a lower frequency in the action stages compared to patients in the no pica group. Noteworthy, most of patients reported themselves in the action and maintenance stages regarding tobacco and alcohol consumption without differences between groups.

Regarding lifestyle evaluation (Table 2), it was observed that patients with hard pica had lower scores in all dimensions (and consequently in the total score), except for tobacco and alcohol consumption, compared to patients without pica. Similarly, patients with ice pica scored significantly lower compared to the no pica group in total score, and in the emotion management and adherence to treatment dimensions. The hard pica group also had lower knowledge of disease score compared to patients with ice pica.

**Table 2:** Comparison of healthy lifestyle domains scores according to the presence of pica.

**Lifestyle dimension**	**Max. score**	**No pica (*n* 233)**	**Ice pica (*n* 78)**	**Hard pica (*n* 89)**
Diet	36	26.8 ± 4.9	25.6 ± 3.8	23.9 ± 6.3^*^
Physical activity	12	7.0 ± 3.3	6.1 ± 3.1	5.5 ± 3.4^*^
Tobacco	8	7.7 ± 1.0	7.8 ± 0.9	7.8 ± 0.9
Alcohol	8	7.7 ± 1.0	8.0 ± 0.4	7.8 ± 0.4
Knowledge of disease	8	6.4 ± 2.0	6.6 ± 2.0	5.7 ± 2.7^*&^
Emotion management	12	8.0 ± 3.7	6.7 ± 4.0^*^	6.6 ± 3.9^*^
Adherence to treatment	16	14.7 ± 2.0	13.7 ± 3.3^*^	13.4 ± 2.9^*^
Total score	100	78.5 ± 9.8	74.5 ± 8.9^*^	70.1 ± 11.5^*^

^*^p <0.05 vs. no pica.

^&^p <0.05 vs. ice pica.

Analysis of food consumption frequency is shown in Table 3. Compared to patients with no pica, those with hard pica ate vegetables and fruits less frequently, whereas they ate dairy products, fried foods and soda more frequently, although the latter was significantly different only compared to patients with ice pica. Patients with ice pica consumed less frequently fish and more frequently processed meat compared to patients in the no pica group. No differences were observed between groups in legumes, tortillas, bread, pork, beef meat, chicken, egg and sugar intake frequency.

**Table 3:** Comparison of food consumption frequency according to the type of pica.

**Variable**	**No pica (*n* 233)**	**Ice pica (*n* 78)**	**Hard pica (*n* 89)**
**Plant–based foods**			
**Vegetables**, ***n*** **(%)**			
Daily	107 (46)	35 (45)	26 (29)^*^
2–3 times per week	90 (39)	32 (41)	48 (54)^*^
2–3 times per month	34 (15)	11 (14)	13 (15)
Rarely/never	2 (0.9)	0	2 (2)
**Fruits**, ***n*** **(%)**			
Daily	165 (71)	47 (60)	40 (45)^*^
2–3 times per week	41 (18)	23 (29)	32 (36)^*^
2–3 times per month	27 (12)	8 (10)	15 (17)
Rarely/never	0	0	2 (2)
**Legumes**, ***n*** **(%)**			
Daily	27 (12)	9 (11)	12 (13)
2–3 times per week	113 (48)	34 (44)	38 (43)
2–3 times per month	58 (25)	18 (23)	28 (31)
Rarely/never	35 (15)	17 (22)	11 (12)
**Tortilla**, ***n*** **(%)**			
Daily	203 (90)	70 (91)	82 (93)
2–3 times per week	20 (9)	5 (6)	6 (7)
2–3 times per month	1 (0.4)	2 (3)	0
Rarely/never	2 (0.9)	0	0
**Bread**, ***n*** **(%)**			
Daily	23 (19)	8 (22)	9 (18)
2–3 times per week	51 (43)	19 (51)	17 (34)
2–3 times per month	36 (30)	9 (24)	19 (38)
Rarely/never	9 (8)	1 (3)	5 (10)
**Animal–based foods**			
**Dairy products**, ***n*** **(%)**			
Daily	64 (27)	19 (24)	19 (21)
2–3 times per week	87 (37)	40 (51)	52 (58)^*^
2–3 times per month	56 (24)	15 (19)	16 (18)
Rarely/never	26 (11)	4 (5)	2 (2)^*^
**Pork**, ***n*** **(%)**			
Daily	16 (12)	5 (10)	4 (7)
2–3 times per week	38 (28)	12 (24)	9 (15)
2–3 times per month	44 (33)	12 (24)	21 (35)
Rarely/never	37 (27)	21 (42)	26 (43)
**Fish**, ***n*** **(%)**			
Daily	4 (3)	1 (3)	0
2–3 times per week	49 (42)	7 (19)^*^	19 (35)
2–3 times per month	31 (26)	21 (57)^*^	19 (35)
Rarely/never	33 (28)	8 (22)	16 (30)
**Beef meat**, ***n*** **(%)**			
Daily	2 (2)	3 (8)	2 (4)
2–3 times per week	76 (61)	22 (59)	33 (60)
2–3 times per month	33 (26)	5 (13)	17 (31)
Rarely/never	14 (11)	7 (19)	3 (5)
**Chicken**, ***n*** **(%)**			
Daily	75 (37)	16 (25)	22 (27)
2–3 times per week	107 (53)	41 (63)	49 (60)
2–3 times per month	13 (6)	5 (8)	7 (8)
Rarely/never	6 (3)	3 (5)	4 (5)
**Egg**, ***n*** **(%)**			
Daily	12 (10)	1 (3)	2 (4)
2–3 times per week	68 (57)	23 (62)	31 (57)
2–3 times per month	24 (20)	8 (22)	18 (33)
Rarely/never	15 (13)	5 (13)	3 (6)
**Unhealthy foods**			
**Processed meat**, ***n*** **(%)**			
Daily	2 (1)	0	0
2–3 times per week	42 (18)	14 (18)	16 (18)
2–3 times per month	77 (33)	39 (50)^*^	37 (42)
Rarely/never	111 (48)	25 (32)^*^	36 (40)
**Fried foods**, ***n*** **(%)**			
Daily	0	1 (3)	2 (4)
2–3 times per week	5 (4)	3 (8)	8 (16)^*^
2–3 times per month	38 (33)	9 (24)	16 (32)
Rarely/never	72 (63)	24 (65)	24 (48)
**Sugar**, ***n*** **(%)**			
Daily	47 (20)	7 (9)	15 (17)
2–3 times per week	98 (42)	42 (54)	44 (50)
2–3 times per month	63 (27)	20 (26)	24 (27)
Rarely/never	25 (11)	9 (11)	5 (6)
**Soda**, ***n*** **(%)**			
Daily	14 (6)	2 (3)	12 (14)^&^
2–3 times per week	23 (10)	13 (17)	11 (12)
2–3 times per month	61 (26)	19 (24)	23 (26)
Rarely/never	135 (58)	44 (56)	42 (48)

^*^p <0.05 vs. no pica.

^&^p <0.05 vs. ice pica.

## 4 Discussion

To our knowledge, this is the first study evaluating the association of the presence of pica with willingness to change lifestyle behaviors and food consumption in patients on dialysis. This study lays the groundwork for this under-evaluated aspect among ESKD patients.

In the current study, pica was found more frequently at younger ages, in those with a lower level of education and a longer duration of dialysis, which is consistent with other studies (11–13). It is possible that consumption of non-nutritive substances is more frequent in younger patients and with a lower educational level due to the lack of concern about its negative consequences. On the other hand, it is not completely clear why patients with ice pica had higher serum CRP; however, the mutual reinforcement between fluid overhydration and inflammation cannot be discarded (21). Overhydration was not investigated in depth in this study, although it was observed that weight was higher in patients with ice pica; inflammation in the presence of pica deserves to be considered in further investigations.

Patients with hard pica displayed lower willingness to change negative habits in diet and exercise behaviors than those without pica. The willingness to take actions for the control of medical conditions is a crucial component for successful self-management; the better readiness to change negative habits has higher self-management practices, dietary problem-solving skills, and lower barriers (22); once patients improved one lifestyle behavior, they may increase confidence in their ability to overcome barriers and achieve healthy lifestyle, increasing the probability to change other unhealthy behavior (23, 24). Interestingly, patients in all groups reported high willingness to change tobacco and alcohol consumption, with more frequent action and maintenance stages. The reason for this latter finding was not specifically investigated nor the temporality of the event (i.e., when the tobacco and alcohol consumption cessation started), but it is possible that being in a transplantation program could be partially implicated.

When comparing the lifestyle scores, patients with pica had lower results in diet, physical activity, knowledge of disease, emotion management and adherence to treatment, besides the overall score, compared to patients without pica. These results are in concordance with those of the willingness to change, as patients with the lowest willingness also had the worst habits in terms of diet and exercise, and all the groups had high scores for tobacco and alcohol consumption reflecting healthy habits in these latter aspects. In adults, pica is associated with pregnancy, intellectual disability, psychotic disorders and recurrent or masked depression (25, 26). The exact nature of the overlapping phenomenology is unclear, although hypotheses include shared neurological pathology (brain volume loss, temporal lobe injury, and neurotransmitter abnormalities), learned behavior, and coping mechanisms (symptoms that begin after stress) (27). Although many of these aspects were excluded in this study, it cannot be ruled out the participation of some of them in a subtle way, for example depression, or alteration in coping skills; this is an issue that deserve further investigation.

The reason why patients with hard pica eat vegetables and fruits less frequently, and dairy products, fried foods and soda more frequently, is not completely clear. Previous studies described the presence of pica and the ingestion of some substances but not of specific foods (14, 28–33). Thus, it was interesting in this study that the presence of pica was associated with frequent ingestion of unhealthy food and decreased ingestion of healthful one.

Adequate nutritional status is ideal for patients on dialysis, as it has been repeatedly shown that protein-energy wasting is associated with increased morbidity and mortality (28). However, achieving this goal is not easy nor frequent due to the intricated etiology of protein-energy wasting in this kind of patients. Diet interventions are very complex, and frequently require multiple individualized dietary restrictions, making more difficult patient's understanding and adherence (29). It has been demonstrated that pica (14) and the unwillingness to change unhealthy patterns are independently associated with a higher prevalence of malnutrition (1). Thus, the confluence of such factors, as well as the ingestion of unhealthy foods requires the participation of a multidisciplinary team, including renal dietitians, psychologists, social workers, and physicians. In addition, selection of foods must consider patient's culture, economy and preferences.

Unfortunately, exercise is not part of the standard clinical care, and is associated with multiple barriers, such as lack of motivation, inadequate equipment and training, fatigue, presence of multiple comorbidities and risks for complications and side effects, psychological alterations, lack of information, among others (30). This could explain why few patients of all groups were in the habit of including physical activity in their routinary life (31); however, such a finding was more apparent in patients with hard pica. Whether these factors are present individually or in conjunction, or in different degree, is not completely clear, but members of the healthcare team should have a more active role recommending physical activity (32, 33).

### 4.1 Limitations

Main limitation of the present work is the cross-sectional design that allow us to establish association but not causality. Therefore, it cannot be established whether pica is cause or consequence of unwillingness to change negative habits, the presence of an unhealthy lifestyle or the consumption of certain foods. Other limitation may be that the frequency of consumption was summarized as group of foods and not detailed with specific foods. For example, beef meat included different types and cuts of meat, which we recognize they are not the same. However, as a first approximation, our results are very interesting and set up the basis for future prospective studies on a topic not deeply addressed before.

In conclusion, the presence of pica was more frequently observed in patients with lower willingness to change their negative habits in the case of diet and exercise, in those who had more unhealthy behaviors in the diet, exercise and emotion management dimensions and lower adherence to treatment, as well as in those who ate less frequently healthful foods and more frequently unhealthy foods.

## Data Availability

The raw data supporting the conclusions of this article will be made available by the authors, without undue reservation.
